# Morbidity management in the Global Programme to Eliminate Lymphatic Filariasis: a review of the scientific literature

**DOI:** 10.1186/1475-2883-6-2

**Published:** 2007-02-15

**Authors:** David G Addiss, Molly A Brady

**Affiliations:** 1 WHO Collaborating Center for Control and Elimination of Lymphatic Filariasis in the Americas, Division of Parasitic Diseases, Centers for Disease Control and Prevention, Mailstop F-22, 4770 Buford Highway, Atlanta, Georgia, 30341, USA; 2 Fetzer Institute, 9292 West KL Avenue, Kalamazoo, Michigan, 49009, USA; 3 Lymphatic Filariasis Support Center, The Task Force for Child Survival and Development, 750 Commerce Dr, Suite 400, Decatur, Georgia 30030, USA

## Abstract

The Global Programme to Eliminate Lymphatic Filariasis (GPELF) has two major goals: to interrupt transmission of the parasite and to provide care for those who suffer the devastating clinical manifestations of the disease (morbidity control). This latter goal addresses three filariasis-related conditions: acute inflammatory episodes; lymphoedema; and hydrocele. Research during the last decade has confirmed the importance of bacteria as a cause of acute inflammatory episodes in filariasis-endemic areas, known as acute dermatolymphangioadenitis (ADLA). Current lymphoedema management strategies are based on the central role of ADLA as a trigger for lymphoedema progression. Simple intervention packages are in use that have resulted in dramatic reductions in ADLA rates, a lower prevalence of chronic inflammatory cells in the dermis and subdermis, and improvement in quality of life. During the past decade, the socioeconomic impact of ADLA and lymphoedema in filariasis-endemic areas has received increasing attention. Numerous operational research questions remain to be answered regarding how best to optimize, scale up, monitor, and evaluate lymphoedema management programmes. Of the clinical manifestations targeted by the GPELF, hydrocele has been the focus of the least attention. Basic information is lacking on the effectiveness and complications of hydrocele surgery and risk of post-operative hydrocele recurrence in filariasis-endemic areas. Data on the impact of mass administration of antifilarial drugs on filarial morbidity are inconsistent. Several studies report reductions in acute inflammatory episodes, lymphoedema, and/or hydrocele following mass drug administration, but other studies report no such association. Assessing the public health impact of mass treatment with antifilarial drugs is important for programme advocacy and morbidity control strategies. Thus, although our knowledge of filariasis-related morbidity and its treatment has expanded in recent years, much work remains to be done to address the needs of more than 40 million persons who suffer worldwide from these conditions.

## Background

Lymphatic filariasis causes a wide range of clinical signs and symptoms, including lymphoedema, hydrocele, lymph scrotum, chyluria, tropical pulmonary eosinophilia (TPE), adenopathy, haematuria, and various manifestations of worms in ectopic sites [1], among others. A major goal of the Global Programme to Eliminate Lymphatic Filariasis (GPELF) is to provide basic care for persons who suffer from the major forms of filariasis-related morbidity, both acute (inflammatory episodes) and chronic (lymphoedema and hydrocele). The objectives of this review are to summarize the scientific basis for morbidity management strategies that have been adopted by the GPELF and to identify priorities for research. Other manifestations of lymphatic filariasis (e.g. chyluria) are not addressed, not because these conditions lack public health significance, but because no coordinated public health approach to address them has been established.

Our understanding of lymphatic filariasis morbidity has evolved considerably during the last 20 years [2-5], and this new understanding has led to the current strategies for morbidity management. The clinical manifestations and factors leading to progression of so-called 'filarial lymphoedema' are similar, if not identical, to those for lymphoedema in non-filariasis-endemic areas. Indeed, given the absence of a diagnostic marker for 'filarial lymphoedema', as well as its multifactorial aetiology [4], some have argued that the use of this term should be avoided. The literature on management of lymphoedema in filariasis-endemic areas is relatively limited; considerably more is known about the pathogenesis, clinical management, and psychosocial impact of 'non-filarial' lymphoedema in Europe, Australia, and North America. Although it is outside the scope of this document to systematically review the literature on lymphoedema and hydrocele from non-endemic areas, we will refer to this literature in passing.

This review is divided into three sections corresponding to the three major clinical manifestations to be addressed: acute inflammatory episodes, lymphoedema, and hydrocele. For each of these clinical entities, the available data are reviewed for the following topics: pathogenesis, epidemiology, economic and social impact, and treatment. A fourth section addresses the impact of mass treatment with antifilarial drugs on all three forms of morbidity.

## Methods

For the first three sections of the paper, we searched the entire PubMed database at the National Institutes of Health through November 19, 2006 using the keywords filariasis, lymphangitis, adenolymphangitis, lymphoedema, and hydrocele and then reviewed these references for relevance to this review. We also included relevant reports from the World Health Organisation (WHO), articles known to us to be in press in peer-reviewed journals, unpublished academic theses, and abstracts published in proceedings of meetings of the British Association of Dermatologists and the American Society of Tropical Medicine and Hygiene. For the fourth section of the paper, we searched on the keywords diethylcarbamazine (DEC), ivermectin, albendazole, and selected all papers that 1) described clinical trials or mass treatment with these drugs for lymphatic filariasis, and 2) included outcomes of hydrocele, lymphoedema, or acute inflammatory episodes. We also included clinical and mass drug trials that had been published before PubMed was established, which we identified from citations and reviews of the earlier literature on DEC in lymphatic filariasis.

## Acute inflammatory episodes (acute attacks)

The aetiology of acute inflammatory episodes in lymphatic filariasis has long been a subject of debate and confusion. Indeed, a variety of terms have been used in the literature to describe them, including 'adenolymphangitis (ADL)', 'acute attack', 'filarial attack', and 'endemic lymphangitis', among others [6]. As early as the 1920s, some scientists argued that bacterial infections were the primary cause of 'filarial' lymphangitis [7-9]. In 1924, the British Filariasis Commission went so far as to state that "all the pathological manifestations" of lymphatic filariasis were caused by secondary bacterial infections [10]. During World War II, clinical and pathologic studies of soldiers with adenolymphangitis and other early clinical manifestations demonstrated the importance of *Wuchereria bancrofti *adult worms or 4^th^-stage larvae [11]. The debate continued after World War II, when the role of the immune system in triggering adenolymphangitis, as well as other forms of filarial pathology, was emphasized [12].

One of the major factors contributing both to the debate and the confusion during the latter half of the 20^th ^century was the relative lack of emphasis on careful clinical observation and case definitions. In 1999, Gerusa Dreyer and colleagues, working in Brazil, defined two distinct clinical syndromes: acute filarial lymphangitis (AFL), caused by death of the adult worm, and acute dermatolymphangioadenitis (ADLA), associated with secondary bacterial infection [13]. AFL is characterized by lymphangitis that progresses distally or in a 'retrograde' fashion along the lymphatic vessel, producing a palpable 'cord'. Rarely, AFL is accompanied by mild fever, headache, and malaise. Distal lymphoedema may occur, but is usually mild and reversible, i.e. self-limited. In contrast, ADLA (a term first used by Olszewski) [14] develops in a reticular or circumferential pattern, and is clinically similar to erysipelas or cellulitis. Symptoms of local pain and swelling, as well as fever and chills, are present. In filariasis-endemic areas, ADLA occurs much more commonly than AFL [13].

Although there is general agreement on the two clinical syndromes as described by Dreyer et al., it has also been suggested that exposure to 3^rd^-stage filarial larvae causes lymphangitis and triggers the onset or progression of lymphoedema. A role for 3^rd ^or 4^th^-stage larvae in lymphangitis or lymphoedema is supported by animal studies, experimental infections [15], reports of disease in individual patients travelling from non-endemic areas [16], and epidemiologic observations that associate incidence of acute adenolymphangitis with filarial transmission intensity [17,18]. However, a case definition has not been established for larva-associated lymphangitis that distinguishes it from AFL or ADLA; this makes epidemiological study difficult. Additional work is needed to clarify the incidence, possible mechanisms, and clinical expression of larva-associated filarial lymphangitis and to assess its public health importance in filariasis-endemic areas.

Recent speculation also has focused on a potential role for *Wolbachia *in the pathogenesis of filaria-related disease [19,20]. Lammie and colleagues have suggested that the pathogenesis of disease in lymphatic filariasis is multifactorial, and have proposed a model that involves the immune system and also allows for a variety of possible causes [3].

Limited attention has been paid to the differences in pathogenesis and clinical manifestations between brugian and bancroftian filariasis. Obvious differences have been noted, such as the absence of male urogenital involvement and chyluria and the much more frequent occurrence of abscesses at the site of lymph nodes in brugian filariasis. However, the reasons for these differences are poorly understood.

### Acute dermatolymphangioadenitis

#### Pathogenesis

Evidence for a bacterial aetiology of ADLA in filariasis-endemic areas comes from the distinctive clinical signs and symptoms, isolation of bacteria at the time of the acute episode, and changes in antibody titres between acute and convalescent serum specimens [21-31]. In India, the bacteria most frequently associated with ADLA are Group A *Streptococcus*. Other bacteria are often found in cultures, including those that are usually regarded as non-pathogenic [22,24,28].

Available evidence indicates that the immune system may amplify or modulate ADLA. The relative infrequency with which bacteria are isolated from patients with ADLA [22,29,32], as well as from persons with cellulitis in areas not endemic for lymphatic filariasis [33,34], suggests a role for inflammatory mediators [33-35], perhaps even in the absence of bacteria.

Little has been published on the antimicrobial sensitivity of bacteria isolated from persons with ADLA in filariasis-endemic areas. Available experience suggests that the organisms most commonly involved are sensitive to penicillin; thus, penicillin is usually recommended for treatment [36,37].

Clinical descriptions of ADLA in filariasis-endemic areas are remarkably similar to those of erysipelas and cellulitis, about which much has been written in the dermatologic literature [38]. Group A *Streptococcus *is the classical causative organism for erysipelas, and lymphoedema is a well-recognized risk factor for erysipelas and cellulitis in areas not endemic for lymphatic filariasis [35].

#### Epidemiology

During the early 1990s, the Special Programme for Research and Training in Tropical Diseases (TDR) sponsored a series of population-based studies on the incidence of 'acute attacks' among the general population in filariasis-endemic areas. The case definition – localized pain, lymphadenitis and/or lymphangitis and/or cellulitis and local warmth, with or without systemic manifestations of fever, nausea, and vomiting (in some studies, lasting for at least three days) – is consistent with ADLA. In these studies, the overall incidence of ADLA ranged from 33 per 1000 per year to 97 per 1000 per year [39-41]. A study from Papua New Guinea, which found an incidence of 31 attacks per 1000 population per year, included only cases with fever [42]. One study in an area endemic for *Brugia malayi*, which also included only cases with fever, found an incidence of 371 episodes per 1000 people per year [43]. Taken as a group, these studies indicate that the rate of ADLA is higher in persons with chronic disease, principally lymphoedema. Among patients in filariasis-endemic areas, the mean annual reported incidence of ADLA ranges from 1.5 to more than 7 episodes per patient [21,32,41,44-51] (Table 1).

**Table 1:** Incidence and duration of acute dermatolymphangioadenitis (ADLA) in filariasis-endemic areas.

**Study**	**Annual incidence of ADLA in general population (per 1000)**	**Annual incidence of ADLA in 'patients' (per patient)**	**Mean duration of ADLA episode (days)**	**Study site**	**Notes**
*Bancroftian filariasis*					

Addiss 1999 [47]	--	2.1^	--	Haiti	
Alexander 1999 [42]	31	--	16	Papua New Guinea	Only cases with fever and ADLA in lower limb
Babu 2005 [48]	85.0	1.6†	3.9	Orissa, India	
Gasarasi 2000 [39]	33	--	8.6	Tanzania	
Gyapong 1996 [40]	95.9	--	5.1	Ghana	
Kanda 2004 [44]	--	1.5^	10.6	Haiti	
Krishnamoorthy 1999 [45]	--	6.4‡	4.1	Tamil Nadu, India	
Kron 2000 [52]	--	--	4.5	Philippines	
Mathieu 2005 [49]	--	2.3^	7.3	Togo	
McPherson 2006 [50]	--	1.6^	--	Guyana	
Pani 1995 [21]	--	4.2^	4.1	Tamil Nadu, India	
Ramaiah 1996 [41]	96.5	1.8†	3.6	Tamil Nadu, India	
Sabesen 1992 [46]	49.8	6.0^	3.9	Tamil Nadu, India	

*Brugian filariasis*					

Pani 1989 [51]	--	4.9^ 7.6^	4.9 5.8	Kerala, India	Stage 1 oedema Stage 2 oedema
Rao 1982 [43]	371*	--	1.4	Kerala, India	Only cases with fever
Sabesen 1992 [46]	41.4	5.4	4.9	Kerala, India	
Suma 2002 [32]	--	4.7^	--	Kerala, India	Restricted to patients with ≥ 2 ADLA episodes.

^Lymphoedema patients only

‡ Lymphoedema and hydrocele patients

† Among persons with one or more ADLA episodes in 1-year observation period

*Calculated from 7 month follow-up

The duration of ADLA, primarily based on patient self-reporting, ranges from 1 to 16 days [21,32,39-46,48,49,51,52] (Table 1). Recurrent ADLA episodes result in significant short-term disability, and are of much greater concern to patients than is lymphoedema *per se *[53]. Studies in Ghana indicate that patients with ADLA are incapacitated for 3 of the 5.1 days of ADLA duration [40]; in Tanzania, patients are incapacitated for 3.7 of the 8.6 days of ADLA [39]. In India, total disability from ADLA lasted no more than 3 days in an area with brugian filariasis [43]. However, preliminary data from Haiti [44] and Togo [49] suggest that the number of workdays lost may exceed the duration of the acute ADLA episode itself.

Among persons with lymphoedema, risk factors for ADLA include increasing patient age [39-41], poor hygiene [54], and illiteracy [47]. Gender, lymphoedema severity, and the presence of entry lesions are additional risk factors. Females tend to experience higher rates than males, although exceptions have been noted [41]. The relationship between lymphoedema stage and incidence of ADLA is not consistent among all studies. It is complicated, in part, by the use of different systems to stage lymphoedema. Most studies show a positive association between lymphoedema stage and observed or patient-reported incidence of ADLA [6,13,32,55-58]. However, other studies – all of which relied on patient recall of ADLA incidence – found no such association [39,40,44,45]. Data from Brazil, India, and Guyana indicate that the presence [54] and number [50,59] of interdigital skin lesions are remarkably strong risk factors for ADLA.

The epidemiologic association between ADLA frequency and stage, as well as extensive clinical experience from both filariasis-endemic and non-endemic areas, strongly suggest that ADLA episodes are a major – likely the most important – factor in lymphoedema progression, particularly in filariasis-endemic areas.

#### Economic and psychosocial impact

##### Cost

Studies from India, Ghana and Haiti indicate that ADLA treatment costs to patients range from US$ 0.25 to US$ 1.62 per episode, as much as two days' wages [44,45,60-63]. In Sri Lanka, Chandrasena reported costs of US$ 7.38 per episode for care from private practitioners, although most patients received free treatment at government clinics [64]. These costs included direct costs of treatment, including self-medication, as well as travel. Two studies also included costs of food and accommodation [45,61]. In all cases, except for consultations with herbalists in Haiti, patients seeking care from health centres or private providers spent more money than those seeking care from traditional practitioners, primarily because these providers had higher consultation charges. In addition, payment was often provided in-kind when care was given by members of the extended family or traditional practitioners. At the upper end of the spectrum, Kron et al. calculated costs for personal expenses in the Philippines as high as US$ 25 per ADLA episode, excluding lost wages [52].

##### Productivity

Much of the burden of ADLA comes not from treatment costs, but from indirect costs due to lost productivity. ADLA episodes significantly affect patients' abilities to carry out both economic (farming, market activities, building) and domestic (household chores, cooking, taking care of children) activities [39,40,43,44,53,64,65]. ADLA episodes are more disabling than other febrile illnesses [61,66]. This incapacitation results in productivity losses; studies in India and Tanzania showed that patients with ADLA spent an average of 2.7–3.6 hours less per day on economic activities than controls [39,60,63,66].

Studies indicate that ADLA episodes reduced potential community labour supply in Ghana by 0.79% [61] and in Indian communities by approximately 0.1% [63,66]. While these figures represent a much smaller loss than that from chronic filarial disease (7% of potential labour lost), they do not adequately capture the impact of ADLA at the level of the household. Household-level effects, including time lost from work and school for caregivers, have not been studied in detail.

Even with these modest estimates, the productivity lost due to ADLA represents a significant loss of potential income. Sabesan estimated that US$ 160000 per year is lost to ADLA among persons with lymphatic filariasis in Pondicherry, India [46], while other studies in India estimate a national figure of US$ 60–85 million lost per year [45,67]. Kron estimated that US$ 38 million is lost annually due to ADLA in the Philippines [52].

##### Quality of life

Several studies have reported a strong negative effect of ADLA on quality of life [53,68-73]. A study of patients at a filariasis clinic in Haiti found that ADLA affected several quality of life indicators, including how much one thinks about the disease and the ability to work [53]. A qualitative study in the Dominican Republic found that the greatest physical and psychological distress occurs during ADLA, regardless of stage of lymphoedema (B. Person, personal communication). Ninety-six per cent of women interviewed in this study described distress not only from the pain and disability caused during the ADLA episode, but also from anticipation of future episodes. A study of the effect of lymphatic filariasis on schoolchildren in India found that ADLA led to frequent absenteeism and impaired performance [68].

In another recent study, patients in India ranked ADLA higher than lymphoedema and hydrocele in terms of severity, with an average severity score of 25–27 on a scale of 0–28. Patients also cited 'very severe problems' in the domains of mobility, self-care, usual activities, pain, anxiety/depression and social participation on an extended EuroQol scaling system [69]. They reported curtailing their activities and interactions with others in an attempt to prevent future ADLA attacks from occurring. Other studies have noted the pain, restrictions and dependency that result from ALDA episodes, but have not translated this into standard quality-of-life indicators [71,72].

##### Health-seeking behaviour

Studies in India found that 49%–98% of lymphoedema patients sought treatment for ADLA during the previous 6–12 months, either by consulting government or private health personnel or self treating. Patients in urban areas were more likely to seek treatment [45,60,62,63]. In a study in rural Haiti, approximately 50% of people experiencing an ADLA episode sought treatment from health clinics, traditional healers, or by self-treating [44]. In rural Ghana, Gyapong et al. found that 55% of those suffering ADLA episodes sought care (with only 1% going to government health facilities), compared to 88% of those with other febrile illnesses. Because of distance to health facilities, difficult terrain, and the pain associated with ADLA, many patients do not seek treatment outside the home until the episode is almost over [61,74]. In addition, many patients believe that ADLA is not preventable, since it recurs even with treatment, so they stop seeking treatment [60,63,74,75]. Data from Togo confirm this impression, and indicate that many patients have sought help in the past for ADLA from a wide variety of sources, but currently either self-medicate or do not seek help [49]. Traditional practices for ADLA include herbal preparations which are smeared on the affected limb, scarification or cutting the skin, and analgesics bought from local drug peddlers [61,72,75,76].

#### Treatment and prevention

##### Treatment

Treatment recommendations for ADLA include rest, cooling the affected area to relieve pain and limit thermal-related damage to the skin, analgesics and antipyretics to relieve pain and fever, systemic antibiotics, and elevation of the affected limb [27,36]. Little is known about the degree to which antibiotics shorten the duration of ADLA episodes, but as with erysipelas and cellulitis in areas not endemic for lymphatic filariasis [38], antibiotic treatment is recommended [36,37].

##### Prevention

*Basic lymphoedema management*. An increasing number of studies have documented the effectiveness of basic lymphoedema management, as recommended by WHO, in reducing the incidence of ADLA episodes [47,64,77][78,79]. In Guyana, McPherson found that 10 of 11 patients had reported ADLA during the six months preceding enrolment in a hygiene education programme, compared to none of them during the six months after enrolment [78]. A recent evaluation by WHO reported dramatic reductions in incidence of ADLA in Sri Lanka, Zanzibar (United Republic of Tanzania), and Madagascar [77]. In India, several placebo-controlled studies have observed significant decreases in ADLA incidence among lymphoedema patients who only received instruction in foot care [30,55,56].  

Reductions in ADLA frequency can be maintained for several years through home-based care.  In Haiti, the reported incidence of ADLA during the year before beginning treatment was 2.1 episodes per year; this decreased to 0.6 episodes after hygiene and skin care were emphasized [47]. A follow-up assessment 18 months after the patients ‘graduated’ from clinic visits, but continued lymphoedema care at home, showed an annual incidence of 0.5 ADLA episodes per year [53]. Suma and colleagues reported sustained practice of self-care among patients in an area endemic for brugian filariasis; some two years after patients had received ‘foot care’ education, 95.3% reported having fewer or less severe ADLA episodes, with a mean incidence of 2.8 acute attacks per year [32].  

*Prophylactic antibiotics*. For patients who continue to experience frequent episodes of ADLA despite basic measures of hygiene and skin care, prophylactic antibiotics are recommended [36]. This practice is also recommended in non-endemic countries for patients with lymphoedema who have recurrent cellulitis. The effectiveness of prophylactic antibiotics has been evaluated in several studies. Olszewski examined the effect of benzathine penicillin, given at three-week intervals for one year, on the incidence of ADLA, and reported a dramatic decrease, with recurrent episodes occurring only in 9% of patients [14]. In a placebo-controlled trial in Vellore, India, lymphoedema patients who received prophylactic penicillin experienced greater decreases in ADLA incidence than those who only received training in foot care [30]. However, in similar studies in Kerala, India, Shenoy and colleagues found that, for most patients, antibiotics provided little additional benefit if foot care was regularly practiced [26,55,56]. Kerketta and colleagues, in Orissa, India, observed lower rates of ADLA among patients who were randomized to receive foot care and penicillin prophylaxis than among patients not receiving penicillin, although the difference was not statistically significant [79]. A recent Cochrane review concluded that although penicillin and foot care appear to reduce the frequency of cellulitis, further studies are needed to document the effectiveness of these measures [80].  

*Antibiotic soap*. An unpublished study from Haiti found that the incidence of ADLA in lymphoedema patients decreased to a similar extent (from 1.1 episodes to 0.4 episodes per year) in patients who washed with antimicrobial soap and those who received standard soap [58], suggesting that hygiene itself was more important than the antimicrobial content of the soap.    

*Participation in patient support groups.* Participation in patient support groups has been shown to decrease the number of ADLA episodes and improve quality of life among lymphoedema patients in Haiti [81].  

*Risk of death*. Fatal outcomes for ADLA are thought to be uncommon, but most programme managers and clinicians who care for patients with lymphoedema are aware of at least a few cases in which ADLA progressed to septicaemia and death. The actual incidence of fatal outcomes with ADLA is unknown, and risk factors for severe or fatal ADLA are poorly characterized.  The clinical experience of Dreyer and others indicates that elderly patients, alcoholics, and patients with malnutrition, hypertension, diabetes, or chronic cardiac or pulmonary disease may be at increased risk of severe ADLA [36].  

### Acute filarial lymphangitis

#### Pathogenesis

As noted above, among persons born and raised in areas endemic for bancroftian filariasis, episodes of AFL, due to death of the adult worm or 4^th^-stage larva, are less severe and have less systemic involvement than ADLA. Systemic involvement may be greater in 'immune-naïve' immigrants to endemic areas. Classical AFL was described extensively in US and European soldiers during World War II [11,82-85]. AFL is commonly observed following individual or mass treatment with DEC [86,87], and this is considered evidence of the drug's macrofilaricidal efficacy [88-90].

#### Treatment

Treatment of AFL is supportive. Cold compresses, rest, and analgesics are recommended. Treatment with antifilarial drugs during acute inflammatory episodes used to be recommended, but now is not considered indicated [27,36,91].

#### Acute filarial lymphangitis and clinical disease

The degree to which AFL triggers or hastens the development of hydrocele in bancroftian filariasis has been investigated by several authors. Norões and colleagues reported a 22% incidence of acute hydrocele following a single 'scrotal nodule event', whether spontaneous or induced by DEC [92]. Overall, 5% of men with scrotal nodules (adult worm death) developed hydrocele that persisted for 18 months or longer. Similar findings were observed in Haiti following mass treatment with DEC and albendazole [93]. Hussein and colleagues in Egypt found that 14 of 16 infected men developed detectable fluid in the tunica vaginalis cavity after treatment with DEC and albendazole, of whom three developed chronic hydrocele [94]. It is unclear whether the lifetime risk of acute or chronic hydrocele is increased by DEC treatment, or whether the drug merely synchronizes adult worm death and, therefore, resulting hydrocele.

AFL appears to trigger the onset of lymphoedema less frequently than it does hydrocele, and persistent lymphoedema following AFL is unusual in the absence of other co-factors [5].

## Lymphoedema

The literature on lymphoedema in filariasis-endemic areas suffers from a lack of standardization, terminology, and agreed-upon criteria for diagnosis and case definition. Indeed, many authors use the term 'elephantiasis' for all forms of lymphoedema. Further, even in non-endemic areas, there is no one system for classifying or staging lymphoedema that is universally accepted [95]. The lack of standardization limits our understanding of the epidemiology, prevalence, and severity of lymphoedema. Further, the prevalence of co-morbidity, especially venous disease, associated with lymphoedema in filariasis-endemic areas is unknown. An urgent need exists for standardization of terms and common case definitions, and for improved knowledge about co-morbidity and its effect on recommended treatment practices.

### Pathogenesis

The pathogenesis of lymphoedema in filariasis-endemic areas has been a matter of intense debate. For many years, it was believed that a shift in antifilarial immunity triggered the onset of lymphoedema, before which time the asymptomatically infected host was 'in harmony' with the parasite [96,97]. However, clinical observations and ultrasonographic and lymphoscintigraphic examinations demonstrated that lymphatic vessel dilatation and dysfunction commonly occur in the absence of lymphoedema. The molecules or processes that stimulate lymphatic vessel dilatation, and the mechanisms by which this process is maintained, are unknown. The clinical model proposed by Dreyer emphasizes that lymphoedema in filariasis-endemic areas is a multifactorial process [4].

Alternative models have been proposed. Epidemiologic associations between transmission intensity and the prevalence of lymphoedema have suggested to some investigators that third-stage larvae trigger lymphoedema [18,98]. This hypothesis is supported by observations of decreases in lymphoedema prevalence and severity following mass treatment with antifilarial drugs [18]. Although such reductions are not always observed, these findings suggest that mass drug administration could have therapeutic benefits on filarial morbidity.

Longitudinal studies showing that asymptomatic microfilaraemic persons are less likely than uninfected persons to develop lymphoedema suggest an immunologic mechanism [99]. Recent studies also have suggested a possible role for *Wolbachia *in the pathogenesis of lymphoedema [19,20].

### Epidemiology

Globally, an estimated 16 million persons suffer from lymphoedema in filariasis-endemic areas of the world [100]. Clinically, so-called filarial lymphoedema is often indistinguishable from lymphoedema of other causes, and there is no laboratory marker that proves, at the individual level, that the initial (or only) cause of lymphatic vessel dysfunction was damage associated with adult filarial worms.

The earliest onset of lymphoedema in filariasis-endemic areas is usually observed around the time of puberty, and the prevalence increases with age [101-103]. In many areas where bancroftian filariasis is endemic, lymphoedema of the leg is more common in women than in men [104-106], although this finding is not universal [103], especially in areas with brugian filariasis [102]. Gyapong and colleagues have reported an association between the community prevalence of lymphoedema and that of microfilaraemia [107].

Little is known about what triggers the onset of clinical lymphoedema in filariasis-endemic areas, or about what factors cause lymphoedema, once triggered, to persist. After lymphoedema is established, recurrent episodes of ADLA are thought to be the major factor associated with disease progression, although the role of other factors remains largely unexplored. Scarification of the skin, a traditional practice in many filariasis-endemic areas, is considered a risk factor for rapid progression of filarial elephantiasis because of the increased risk of ADLA [76].

### Economic and psychosocial impact

#### Cost

Costs to patients for lymphoedema treatment, reported as both per-visit and per-year costs, vary greatly by study. A study in India reported an average of US$ 0.56 per visit, more than half a day's wages [62]. A Ghanaian study reported costs for treatment of chronic disease (both lymphoedema and hydrocele) of US$ 0.87 per visit, equivalent to almost one day's wages [61], and greater than costs incurred by controls with other chronic diseases. In India, the annual cost for lymphoedema treatment ranges from US$ 2.17 to US$ 8.70 per person [108,109]. Average treatment costs are often low, in part because many patients who find potential treatment costs prohibitive either self-treat or do not seek treatment [109-111].

#### Productivity

Productivity losses from lymphoedema have been captured as lost working hours and as changes in individual output. Lymphoedema patients in India lose 0.55 to 1.61 hours per day in time at work; 11%–31% of workdays are lost annually [66,109]. These findings are similar to those of another study of both lymphoedema and hydrocele patients, which estimated 1.13 hours lost per day, for a total of 19% of workdays lost per year [108]. In Ghana, female labour input loss due to lymphoedema was estimated at 1.5% per year, using the average percentage of lymphoedema patients unable to complete certain activities and the local prevalence of lymphoedema [61]. In general, many patients report changing to less strenuous occupations or giving up working altogether due to lymphoedema and ADLA [69,72,75,112]. A study of male weavers in India with chronic disease, 26% of whom had lymphoedema, found a 27% decrease in output compared to controls [113].

#### Quality of life

Several studies have quantified the impact of lymphoedema on quality of life using standardized measures [44,69,70,73,78,114,115]. McPherson, using a 30-point Dermatology Quality of Life Index in Guyana, found a mean baseline score for lymphoedema patients of 10.9 (comparable to patients with psoriasis and atopic eczema in the United Kingdom), with controls scoring 0.5 [83]. Six months after starting regular hygiene treatment, the scores improved significantly by an average of 6.8 points [122]. In Haiti, Kanda compared different ways of measuring quality of life among rural people with lymphoedema [46]. Using the EuroQol scale, he found that no respondents had extreme problems in mobility or self-care, but more than half reported pain or discomfort. On a depression scale, the CES-D, these same patients had a mean score of 13.2 (16 and above indicates depression). On the CDC Healthy Days questionnaire, Kanda found that 88% of patients ranked their health as fair or better; however, they also reported an average of 9.9 physically or mentally unhealthy days during the past month. Advanced age, advanced stage of illness, and low educational level were strongly associated with lower quality-of-life measures [44]. In India, patients with lymphoedema scored from 9.2 to 12.4 on a 28-point scale of 'health state severity' using an extended EuroQol measuring system [69]. Severity was associated with stage of lymphoedema; in higher stages, 'severe or very severe problems' were reported for the domains of usual activities, pain, anxiety/depression, cognition and social participation. Among men, the severity score for lymphoedema was significantly higher than that for hydrocele [70].

##### Stigma

Many studies mention the stigma surrounding lymphoedema, but they differ in the severity of stigma reported. Diminished marriage prospects and/or threat of divorce due to diminished economic productivity and attractiveness are often cited as problems for persons with lymphoedema, both by the patients themselves and by other community members [65,69-75], [116-119]. This effect appears to be dependent on age of lymphoedema onset and disease stage [71]. In Haiti, patients reported that their children had the most difficulty coping, as they were often teased or embarrassed about the mothers' lymphoedema [71]. Following a series of 'soap opera' radio broadcasts in Haiti, which were intended to decrease social stigma associated with lymphoedema, patients reported improved self-efficacy and social support [120].

##### Impact on activities

A study in Ghana, which did not distinguish between lymphoedema and hydrocele, found that those with chronic filariasis were significantly less likely to be able to perform market and building activities than matched controls [61]. Among patients in India who were visited at home during the course of a year, those with chronic filarial disease were found to be totally incapacitated at 22% of visits, compared to 13.4% for controls, a significant difference [108]. Another study in India found that lymphoedema patients reported a negative impact on domestic activities (15%–33% of patients), economic activities (65%–83%), and movement (67%–78%) [121]. Lymphoedema patients in Haiti reported decreased ability to walk, difficulty in finding appropriate footwear, and sometimes inability to sell at the market or do household chores [71]. Among those practicing lymphoedema self-care, 25% stated that lymphoedema limited their ability to work [53].

##### Emotional impact

Among filariasis clinic patients in Sri Lanka, 18% felt they were being shunned by society, although these data were collected after the patients had been enrolled in treatment [64]. In other studies, almost all patients report negative feelings of frustration, isolation, or embarrassment resulting from their condition or their inability to find effective treatment [53,71-74,116,120,122,123]. As lymphoedema progresses, the negative emotional and psychological impact often worsens. Patients in an Indian study expressed suicidal thoughts [69] and depression was common among patients in Haiti and Togo [44,49]. Anecdotal reports from other filariasis-endemic countries suggest that suicidal ideation and depression are not uncommon among persons with lymphoedema.

##### Social support

A study in Haiti of patients enrolled in a lymphoedema treatment clinic found that the odds of regularly practicing hygiene and skin care were 3.7 times greater among patients who believed that family members supported them than among those who didn't mention family member support [120]. Participation in patient support groups was shown to decrease the number of ADLA episodes and improve quality of life among lymphoedema patients in Haiti [81]. In Brazil, patient 'Hope Clubs' have been developed to provide ongoing opportunities for social and emotional support, problem-solving, and continued learning [124].

#### Health-seeking behaviour

Studies in India and Ghana show that 46%–100% of persons with lymphoedema sought treatment from health care centres, local healers, or pharmacies during the previous year [61,62,108,109]. The studies in Ghana show that modern medical care often is avoided due to lack of interest from health care workers and a belief by patients that lymphoedema treatment requires spiritual interventions [74,118]. Although many patients believe lymphoedema progression cannot be prevented, they continue to consult spiritualists and treat themselves with herbal preparations or analgesics [75]. In contrast, in areas of India with networks of public healthcare facilities, most patients seek care from modern medical practitioners, although a minority consult Ayurvedic doctors or use home remedies first [72,111]. Access to care is not necessarily universal, however; young women in India may not seek treatment because of social constraints, such as the paucity of female doctors [116]. Other barriers to care include distance to a health facility, lack of awareness, lack of time, lack of child care, perceived severity of disease, and dissatisfaction with previous treatment [74,116,122,123]. Even when patients seek treatment, health personnel often will prescribe antifilarial or other drugs that are expensive and ineffective. Inadequate knowledge of lymphoedema management by health workers results in suboptimal patient care [105,111,123,125,126].

### Beliefs and traditional practices

Beliefs about the cause of lymphoedema include heredity, supernatural and spiritual causes, and natural causes such as injury, standing in cold water, stepping on insects, and ingesting unhygienic food or drinks [52,71,74,75,110,116,119,122,127-131].

In filariasis-endemic areas, people with lymphoedema seek help from traditional healers, herbalists, sorcerers, and pharmacies, or they self-treat. Traditional treatment for lymphoedema includes herbal preparations, burial of the leg, scrubbing the surface of the foot with ants, bloodletting, and scarification, among others [71,74,76,110,119,122]. Even in areas with established clinics for lymphoedema management, where patients have learned the importance of hygiene, skin care, elevation and proper footwear, many still hope for a permanent cure [64,71,120,132] (B. Person, personal communication).

### Treatment and prevention

Recognition of the importance of ADLA in the progression of lymphoedema has led to basic recommendations for the treatment of lymphoedema in filariasis-endemic areas. The cornerstones of this treatment include hygiene, skin care (early detection, treatment, and prevention of entry lesions), exercise, and elevation of the affected limb [27,36,133]. In addition to the above measures, appropriate footwear is recommended, and prophylactic antibiotics are recommended for some patients.

All of these recommendations are consistent with proper lymphoedema care in developed countries where lymphatic filariasis is not endemic [134,135]. However, in these areas, additional modalities are also used, including compressive bandages, compressive garments, and manual lymphatic drainage [134-137]. These and other measures would no doubt be helpful for individual patients in filariasis-endemic areas [133], but require more training, experience, and resources, and are therefore not included in the public health approach to managing lymphoedema adopted by the GPELF for filariasis-endemic countries [37].

#### Effectiveness of treatment on acute dermatolymphangioadenitis

Relatively few studies have documented the effectiveness or impact of the basic package of lymphoedema management, and most of these have focused on ADLA. The available data indicate that such treatment is associated with a marked reduction in incidence of ADLA [30,32,47,55,56,58,78]. An unpublished study from Haiti reported that risk factors for continued ADLA include more advanced disease, 'negligence', and illiteracy [47].

#### Effectiveness of treatment on leg volume

A few studies have documented changes in leg volume or circumference in response to basic lymphoedema management. Although an 'objective' measurement, leg volume can vary considerably with time of day, exercise, elevation, and other factors. In Orissa, India, Kerketta and colleagues reported significant reductions in leg circumference with all treatment regimens that included basic foot care [79]. Pani and colleagues reported greater volume reductions in patients with oedema of recent onset than in those with lymphoedema of longer duration [51]. An unpublished study from Haiti, which initially included compressive bandaging as one of its modalities, reported that more than 65% of 178 patients had a reduction in leg volume after two years when compared with pre-treatment measurements [47].

#### Effectiveness of treatment on entry lesions

It is commonly observed that, with basic lymphoedema management, the prevalence and severity of entry lesions decrease [36].

#### Effectiveness of treatment on odour

Reduction in offensive odour is commonly observed with regular hygiene. To our knowledge, there have been no studies focusing on reduction in odour as an outcome of lymphoedema treatment in filariasis-endemic areas, although anecdotally this improvement has an important effect on quality of life.

#### Effectiveness of treatment on stage of lymphoedema

Few studies have attempted to address the degree to which basic lymphoedema management results in regression of lymphoedema stage or grade. In part, this is because most staging systems have not been developed for this purpose. Thus, considerable improvement in skin condition or even leg volume is possible without regression in stage *per se*.

#### Effectiveness of treatment on limb flexibility and range of motion

Improved flexibility and a feeling of 'lightness' are commonly reported by patients, but few studies have documented the effectiveness of basic lymphoedema management on limb range of motion.

#### Effectiveness of treatment on quality of life

Several studies are currently underway that address the extent to which basic lymphoedema management in filariasis-endemic areas improves quality of life. One study, by McPherson in Guyana, documented highly significant improvement in quality of life as measured by the Dermatology Quality of Life Index [78]. Similar work in non-endemic areas has shown substantial gains in quality of life with lymphoedema treatment. Patients who incorporate regular lymphoedema management into their daily routines have reported satisfaction with the results [53,138].

#### Effectiveness of treatment on chronic inflammation

A study in Haiti collected skin punch biopsy specimens from the lymphoedematous legs of 27 patients before and about 12 months after they initiated basic lymphoedema management [139]. Follow-up biopsies showed significant reductions in perivascular mononuclear infiltrate in the superficial dermis (41% decrease in prevalence), in perivascular fibrosis in the deep dermis (58% decrease), and in periadnexal mononuclear infiltrate (53% decrease).

#### Optimization of treatment protocols

Although there is general agreement as to the basic elements of lymphoedema management within the GPELF, considerable regional variation exists in the availability of supplies, including soap, water, and topical skin preparations (e.g. antiseptics, antifungal and antibacterial agents). These differences contribute to variation in approaches used in different regions. For example, in some countries, macerated interdigital lesions are treated with Whitfield ointment, an inexpensive antifungal agent, on the presumption that dermatophytes are the primary pathogen. In Guyana, McPherson and colleagues attempted to culture fungi from these lesions and concluded that bacteria probably play a more important role than fungi [50]. McPherson's observations are consistent with studies of intertriginous lesions in non-endemic areas [140,141].

Controlled studies of how best to optimize the effectiveness of treatment, particularly for skin care, have not been published. Some investigators have argued for more widespread adoption of breathing exercises to mobilize lymph fluid, and for emollients to protect and rebuild the skin barrier function [133]. These are issues that are amenable to basic, inexpensive clinical trials.

#### Programmatic challenges

Although there remains some debate about the optimal package of interventions for basic lymphoedema management in filariasis-endemic areas, the benefits of such treatment are generally recognized, and foci of activity in several countries have demonstrated success. However, relatively few persons with lymphoedema living in filariasis-endemic areas currently have access to treatment. Thus, the key programmatic issue is how best to 'scale up' basic lymphoedema management to state and national levels. The challenges can be considered in four major categories:

• Finding patients and bringing them to treatment (many are reluctant to seek care as discussed above)

• Education of patients and family members on the principles and practice of lymphoedema self-care

• Encouragement and support to sustain daily self-care (this support may include improved access to supplies such as clean water, soap, antiseptics, topical antibacterial and antifungal agents, and oral antibiotics)

• Referral networks for management of ADLA and for patients with advanced lymphoedema or lymphoedema complicated by other diseases.

There is general agreement that most patients can manage their lymphoedema routinely at home, and that this is preferable and less costly than clinic-based care. WHO has developed training packages for 'informal caregivers' to instruct patients on home-based care, and this approach has been adopted by most programmes. However, numerous key programmatic and operational research questions remain unanswered for each of the four major programme components. For example: 1) although McPherson and colleagues have shown that health workers in Guyana with limited training can reliably stage lymphoedema and identify entry lesions [142], the ability of such workers to recognize or diagnose lymphoedema in other settings is unknown; 2) the frequency and intensity of education required for patients to become competent in lymphoedema self-care has not been evaluated; and 3) basic requirements for referral care, provider training, and clinical competency have not been determined. The costs of treatment need to be better understood, as well as the benefits. These are areas in urgent need of investigation if the benefits of lymphoedema management are to reach those who most need it.

#### Prevention

Considerable anecdotal evidence suggests that the onset of chronic lymphoedema is triggered by the first or second episode of ADLA. Data from a filariasis-endemic area of Haiti indicate that skin lesions between the toes, which could provide portals of entry for bacteria, are common in children, and are significantly more common in those who test positive for circulating filarial antigenaemia [143]. Similar findings have been observed in northeast Brazil (G. Dreyer, personal communication). The degree to which initial ADLA episodes, and therefore lymphoedema, can be prevented through school-based education programmes focused on hygiene, skin care, and recognition and treatment of entry lesions has not been studied.

## Hydrocele

Despite the greater public health burden of male urogenital disease in lymphatic filariasis, much more attention has been focused to date on management of lymphoedema of the leg. This is beginning to change, as surgery programmes have been launched in several centres. However, many questions remain about diagnosis, optimal management, and cost and benefits of intervention.

### Pathogenesis

In many research papers written during the 1980s and 1990s on the epidemiology or immunology of lymphatic filariasis, all genital swelling in men was labelled as 'hydrocele'. This was in contrast to detailed, even elegant, clinical descriptions of male urogenital disease by investigators in earlier decades [144-147]. Dreyer and colleagues recently emphasized the distinction between lymphoedema of the scrotal and penile skin, which has the same pathogenesis as lymphoedema of the limbs, and swelling due to increased fluid inside the cavity of the tunica vaginalis [36]. This fluid, which is usually considered to be 'hydrocele', actually is comprised of several distinct entities including true hydrocele, chylocele and hematochylocele. The term 'filaricele' has been suggested recently to include all of these manifestations [148].

Norões and colleagues have shown that true filarial hydrocele is triggered by death of the adult worm, which produces an inflammatory nodule that occludes the lymphatic vessel. In this study, the incidence of acute hydrocele following a single 'scrotal nodule event', whether spontaneous or induced by DEC, was 22% [92]. Of these, 24% persist to become chronic. These data are similar to those of ultrasonographic and clinical studies from Egypt [94].

Rupture of lymphatic vessels inside the scrotal cavity can lead to the presence of straw-coloured ('lymphocele') or milky (chylocele) fluid, sometimes with red blood cells. Little is known about the relative frequency of these conditions in different filariasis-endemic areas, and techniques and markers to discriminate among them preoperatively are currently inadequate.

### Epidemiology

An estimated 27 million men suffer from fluid accumulation in the tunica vaginalis in areas endemic for bancroftian filariasis [100]. The prevalence of this condition appears to be strongly associated with intensity of parasite transmission. Gyapong has documented a robust association at the community level between hydrocele prevalence and microfilaraemia prevalence in Ghana [149,150], and this association has been observed elsewhere. The prevalence of hydrocele increases with age.

Little is known about the natural history of hydrocele in filariasis-endemic areas, although increasing (but as yet largely unpublished) evidence seems to suggest that it is much more "fluid" (forgive the pun) than previously realized. Recent observations from Brazil, Egypt, and Haiti indicate that many acute hydroceles resolve spontaneously [92-94].

### Economic and psychosocial impact

#### Costs of non-surgical treatment

Patient expenditures for hydrocele treatment are generally low, as treatment other than surgery is found to be ineffective and most patients cannot afford to pay for surgery. Hydrocele patients in India paid from US$ 1.38 to US$ 4.29 per year for non-surgical treatment; daily wages in the areas studied averaged less than US$ 1.00 [62,108,109]. A Ghanaian study found an average of US$ 0.87 a year (almost one day's wages) spent for treatment of chronic filariasis, which included both hydrocele and lymphoedema – significantly more than was spent by patients with other chronic diseases [61]. In general, treatment costs are difficult to collect accurately as much of treatment is paid in-kind or provided by traditional healers who are members of the extended family.

#### Costs of hydrocele surgery

Published costs to patients for hydrocele surgery range from US$ 5 to US$ 60, depending on the country and source of care. The types of surgery performed and the parameters of costing are not known for all studies. Ramaiah reported costs of US$ 5–14 in government hospitals and US$ 15–47 in private hospitals in India [109], while Babu reported costs of US$ 44 in another Indian study [108]. In Ghana, Gyapong reported surgery costs of US$ 30–35 at local hospitals [61,151] and Ahorlu reported surgery costs of US$ 30–60 for surgery sponsored by non-governmental organizations (NGOs) [152]. Interestingly, the patients in Ahorlu's study estimated that surgery would have cost US$ 75–125 at local hospitals before the NGO programme was put in place. Ahorlu also reported other costs associated with surgery, including transport to hospital and food, estimated by patients at US$ 20–30, with an average hospital stay of 4–12 days.

#### Productivity

Early studies on hydrocele differed in their conclusion about the impact on productivity, and reductions in productivity were not quantified [65,117,129,153]. In recent studies, the effect of hydrocele on productivity has been quantified in three different ways:

• *Individual working hours*. Studies in India have shown that hydrocele patients work approximately one hour less per day than matched controls [66,108,109]. Lu et al. in the Philippines found that 30% of 22 males interviewed lost time from work due to hydrocele [154].

• *Individual output*. A study of weavers with chronic filarial disease in India, 69% of whom had hydrocele, showed that those with disease produced 27% less cloth than matched controls [113].

• *National output*. In India, 8% of potential male labour input was estimated lost due to hydrocele and lymphoedema [109] and this loss was valued at US$ 704 million per year [67]. In Ghana this figure was similar, with more than 7% of potential labour lost [61].

There is almost no evidence on the degree to which hydrocelectomy improves productivity, with only one study reporting qualitative data [152].

#### Quality of life

Early studies described the socially unacceptable nature of hydrocele, but they were vague about the degree of associated stigma, its consistency across communities and cultures, and the psychosocial burden of hydrocele on those affected [65,117,122,155]. To date, research has not been carried out on quality of life in men with hydrocele in filariasis-endemic areas that would allow for comparison with other diseases, or with men who do not have hydrocele.

##### Stigma

Hydrocele patients report both 'enacted stigma' (teasing, problems with marrying and divorce) and 'felt stigma' (ashamed to be part of community activities) [75,152,155]. However, they often develop coping strategies to deal with the stigma [151]. For example, men with hydrocele were less likely to admit that they avoided social events or suffered teasing than were unaffected people to report that they ill-treated men with hydrocele. The severity and visibility of hydrocele, as well as the relationship of patients to community members, seems to correlate with the degree of stigma [117]. Gyapong et al. described general community acceptance of men with hydrocele, but reported that patients with advanced disease often feel ostracized and embarrassed [74]. In Kenya, 36% of men with hydrocele interviewed responded that they were laughed at, while 29%, mostly patients with small hydrocele, reported no reaction from the community [122]. When community members were asked about their reactions to men with hydrocele, those who had family members with hydrocele expressed understanding and sympathy, while others tended to joke about it. In non-endemic villages in Ghana, considerable stigma was associated with hydrocele and lymphoedema, much more than in hyper-endemic villages [118].

##### Impact on activities

In rural India, 8%–10% of men with hydrocele reported a negative impact on domestic work, 53%–55% reported a negative impact on economic activities, and 53%–63% reported decreased mobility [121]. A study in Ghana found that 10%–60% of persons with chronic filarial disease, which included both lymphoedema and hydrocele patients, were unable to perform certain daily activities and were less likely to perform market and building activities than matched controls who had other chronic diseases [61]. Of 14 school-aged boys with hydrocele interviewed in India, one had dropped out of school as a result of being stigmatized and six had high rates of absenteeism [68]. An Indian study measuring the psychosocial and physical burden of hydrocele found that patients' usual activities and social participation were affected by hydrocele, especially for those with larger hydroceles. In addition, as noted in earlier studies [69,155], many men had switched to less demanding occupations as a result of hydrocele.

##### Emotional impact

Men with hydrocele often describe themselves as frustrated, losing hope and even suicidal [117,151,154,156]. In the Philippines, Lu reported that those in higher socioeconomic classes were less emotionally affected as they were aware of, and had access to, surgery [154].

##### Male identity and sexual function

In 1993, the limited literature on hydrocele in filariasis-endemic areas suggested that hydrocele had little impact on sexual activity or fertility [117]. More recently, hydrocele patients in India reported that hydrocele adversely affected their sexual functioning and caused 'moderate problems' with anxiety/depression, based on an extended EuroQol scale [69]. In Ghana, both community members and men with hydrocele reported that hydrocele impeded sexual intercourse, sometimes leading to divorce [75,151]. Qualitative research in Ghana found that men "whose hydrocele interfered with (this) concept of male identity were deeply frustrated"; they felt as if they were a burden to their families because of difficulties in providing for them [151]. In Brazil, Dreyer and colleagues reported several concerns of men with urogenital disease, including genital elephantiasis, which ranged from lack of intimacy in marriage to thoughts of suicide [156].

##### Social support

While perceived social support is important for psychological well-being, we found no published studies that addressed the impact of hydrocele on patients' social support networks or the impact of social support networks on recuperation after surgery.

#### Health-seeking behaviour

A wide range (25%–80%) of hydrocele patients seek treatment [61,62,109,151]. Patients seek treatment from local health centres, traditional healers, and through self-medication. In certain countries, the belief that hydrocele has a supernatural cause leads people to seek out traditional healers or sorcerers instead of modern medical care [74]. While most studies describe treatment only during the previous year, patients may have tried various remedies in the past but stopped seeking care after the treatments were ineffective [74,122,151]. Other reasons for not seeking treatment include problems with access, such as cost of surgery or medical treatment, distance from the health centre, inability to take time off work for recovery, and cultural issues such as fear of anaesthesia during surgery and stigma associated with having hydrocele [61,117,152,154,155]. A study in coastal Kenya found that, in highly endemic districts, hydrocelectomies accounted for 23% of all major operations [157]. Similarly, in Tanzania in 1976, 15% of operations in one district hospital were for hydrocele [158].

### Beliefs and traditional practices

Beliefs about the causes of hydrocele vary by culture and geography, but can be grouped into supernatural causes (including witchcraft and sorcery), heredity, exposure to extreme hot or cold, excessive sexuality, and consumption of certain foods or drinks [74,75,117,122,154]. Some studies also mention hard work or trauma as the cause of hydrocele [127,129,152]. Few mention mosquitoes, even in regions where mass drug administration and health education have occurred [117,127,131,155,159,160]. Only 2.5% of hydrocele patients in a study in rural South India believed that filariasis was transmissible [131]. And the link between filarial infection, hydrocele and lymphoedema is often not understood. Only 1%–4% of people interviewed in an Indian study knew that filarial infection was a major cause of hydrocele [161]. In a study in Orissa, India, while less than half of respondents knew that mosquitoes contributed to the spread of hydrocele, about 70% named them as the cause of lymphoedema [160].

Traditional remedies used to treat hydrocele include herbal preparations, sorcery spells and rites, and draining with hollow reeds [74,75,122]. Perceptions of treatment efficacy vary greatly by region, with a majority of people naming surgery as a cure [74,75,127,155,160]. However, 90% of persons with lymphoedema and/or hydrocele interviewed on the Kenyan coast believed their disease was incurable. This may have been influenced by the experience of two elderly men in the area who had a recurrence of hydrocele after surgery [122].

### Treatment

Surgery is the recommended intervention for hydrocele, and if done properly, it is regarded as curative. Other techniques, such as aspiration of the fluid and injection of sclerosing substances, are less effective, have unacceptable side effects, and have not been adequately evaluated in filariasis-endemic areas [147,162]. Recently, Ryan has called for studies of other measures, such as deep breathing, to reduce the size of hydrocele [163].

A variety of surgical techniques are used for hydrocele in filariasis-endemic areas, although little is known about their relative frequency of use. Modifications of the 'eversion' technique are probably most commonly used, in which part of the tunica vaginalis is excised and the remainder is everted. While this approach may be effective for non-filarial hydrocele or for 'pure' hydrocele in filariasis-endemic areas, it is likely sub-optimal as a procedure for lymphocele or chylocele, because dilated, leak-prone lymphatic vessels – the source of the excess fluid – may not be removed. Thus, the risk of recurrence may be substantial. Eversion techniques have also been associated with other more serious complications, including development of the debilitating condition of lymph scrotum, for which surgical treatment is vastly more challenging than that for hydrocele [144]. Thus, current WHO guidelines call for complete removal of the tunica vaginalis [164].

Few studies have been published on the rates of complications and recurrence following hydrocele surgery in filariasis-endemic areas. A study from Wardha, India, reported a 26.7% incidence of wound infection or haematoma in cases considered filarial in aetiology, compared with 10.9% in non-filarial hydrocelectomies [165]. Among 950 surgeries for large hydrocele in Orissa, India, post-operative infections and abscesses were seen in 28 (2.9%) cases, scrotal haematoma in 12 (1.3%), and reversible penile oedema in 42 (4.4%) [166]. The paucity of data on outcomes makes it impossible to compare cost or effectiveness of one technique over another. Surgical outcomes are currently being evaluated in several countries.

Research is urgently needed to 1) evaluate tools to distinguish the various forms of 'filaricele' preoperatively; and 2) assess costs, resource requirements (e.g. time, anaesthesia, electricity), surgery duration, post-operative quality of life, and incidence of relapse and infectious and other post-operative complications with different surgical techniques.

#### Impact of hydrocele surgery on quality of life

Ahorlu et al. interviewed hydrocele patients in Ghana 1.5 years after surgery. Patients reported that within three to six months post-surgery, they had experienced significant improvement in self esteem, sexual function, and capacity for work, and they participated more in community activities [152].

## Antifilarial drug treatment and filarial morbidity

Data on the impact of treatment with antifilarial drugs on filarial morbidity are inconsistent. Several studies have reported reductions in acute attacks, lymphoedema, and/or hydrocele following mass drug administration, but other studies report no such association (Table 2). For most of these studies, the primary outcome of interest was microfilaraemia rather than clinical morbidity. Therefore, the studies are often limited by inadequate or non-standardized case definitions, inadequate sample sizes, and intermittent or incomplete follow-up. Many studies have only evaluated the effect of drug treatment in persons with existing morbidity. Such an approach ignores the incidence of new cases, and could lead to erroneous conclusions regarding the effect of the antifilarial drugs on disease incidence or prevalence.

**Table 2:** Summary of studies that assessed the effect of antifilarial drug treatment on the clinical manifestations of "acute attacks"*, hydrocele, and lymphoedema.

**Source**	Acute Attacks*	**Hydrocele**	**Lymphoedema**	**Drug**	**Drug delivery strategy**	**Follow-up interval**
Ciferri 1969 [170]	+	--	--	DEC	MDA	2 years
March 1960 [171]	+	+	+	DEC	MDA	10 years
Bernhard 2001 [169]	.	--	.	DEC	MDA, clinical trial	1 year
Partono 1989 [172]	+	.	+	DEC	MDA, selective	11 years
Beye 1952 [173]	--	--	--	DEC	MDA, selective	16 months
Simonsen 1995 [174]	.	--**	.	DEC	Selective	1 year
Kessel 1957 [175]	+	.	.	DEC	Selective	1 year
Fan 1995 [176]	.	--	--	DEC	Salt	16–19 years
Meyrowitch 1996 [177]	.	+	+	DEC	Salt	2 years
Meyrowitch 1998 [178]	.	+	.	DEC	Salt	4 years
Meyrowitch 2004 [179]	.	+¶	.	DEC	MDA, salt	4 years
Hewitt 1950 [180]	+	+¶	+¶	DEC	Clinical trial	8–14 months
Das 2003 [167]	.	.	--	DEC	Clinical trial	1 year
Kenney 1949 [181]	.	.	+	DEC	Clinical trial	1–3 months
Pani 1989 [51]	.	.	+‡	DEC	Clinical trial†	>1 year
Moore 1996 [16]	.	.	+	DEC	Case report	1 week-7 months
Bockarie 2002 [18]	.	+	+	DEC, DEC+IV	MDA	5 years
Dunyo 2000 [182]	.	--	--	IV + Alb	MDA	1 year

* Acute dermatolymphangioadenitis and filarial lymphangitis were not distinguished in most studies

** 2 of 8 hydroceles resolved

¶Disease progression also observed

‡ Reductions seen primarily in patients with early-stage disease

† Included other interventions, but improvement related to number of DEC doses

+ Decrease in size, incidence, or prevalence noted (not necessarily statistically significant)

-- No decrease noted (or if noted, inconsistent or not considered significant by authors)

. Not evaluated or extremely small numbers

DEC Diethylcarbamazine

IV Ivermectin

Alb Albendazole

MDA Mass drug administration using tablets

Salt DEC-fortified salt

Selective Treatment only of persons known to be infected or with clinical disease

Clinical studies have also produced inconsistent findings. A detailed case report of a US Peace Corps volunteer demonstrated dramatic clinical improvement in lymphoedema following DEC treatment [16], but a clinical trial by Das and colleagues in Pondicherry, India found no change in limb volume or condition [167]. A study using lymphoscintigraphy in Recife, Brazil found no improvement in lymphatic morphology among patients with clinical or subclinical disease following treatment with DEC [168]. A carefully designed placebo-controlled study by Bernhard and colleagues found no effect of DEC treatment (in the context of mass treatment) on hydrocele volume [169]. As noted above, treatment with DEC can provoke both acute and chronic hydrocele in men with *W. bancrofti *infection [92,93].

Assessing the public health impact of mass treatment with antifilarial drugs is a critically important issue for programme advocacy and for planning morbidity control strategies. Studies on the impact of antifilarial drugs on the prevalence and incidence of acute inflammatory episodes, lymphoedema, and hydrocele are needed, using rigorous case definitions, close clinical assessment, and control groups. They should be conducted in areas where DEC and albendazole are coadministered as well as in areas where ivermectin and albendazole are used.

## Conclusion

Morbidity control efforts within the GPELF have focused on: 1) basic lymphoedema management (hygiene, skin care, and simple physical measures) to reduce the incidence of ADLA and prevent progression of lymphoedema; and 2) surgical repair of hydrocele. Since the GPELF was launched in 1998, considerable research has documented the effectiveness of basic lymphoedema management and provided a stronger scientific base for this intervention. Less work has been done to document the costs and benefits of hydrocele surgery in filariasis-endemic areas. Additional research is needed to support efforts to 'scale up' morbidity control and disability alleviation programmes at the national level and to document the extent to which antifilarial drug treatment influences the course of filariasis-associated disease.

## Authors' contributions

DGA researched and wrote drafts of the pathology, epidemiology and treatment sections and the section on the effect of antifilarial drug treatment on clinical morbidity. MAB researched and wrote drafts of the economic and psychosocial impact sections. Both authors read and approved the final manuscript.

## Conflict of interest

The author(s) declare that they have no competing interests.
